# Protocol for a parallel-group, superiority randomized controlled trial of the PulsePoint mobile application to increase bystander resuscitation in out-of-hospital cardiac arrest

**DOI:** 10.1016/j.resplu.2025.101036

**Published:** 2025-07-24

**Authors:** Steven C. Brooks, John M. Tallon, Sandra Jenneson, Ashish R. Panchal, Robert Grierson, Laurie J. Morrison, Damon C. Scales, Andrew Day, Lindsay O’Donnell, Randy S. Wax, Helen Connolly, Jennie Helmer, Heidi Corneil, Jim Christenson

**Affiliations:** a Department of Emergency Medicine, Queen’s University, Kingston, Ontario, Canada; b Department of Public Health Sciences, Queen’s University, Kingston, Ontario, Canada; c Department of Emergency Medicine, University of British Columbia, Vancouver, British Columbia, Canada; d Department of Emergency Medicine, Dalhousie University, Halifax, Nova Scotia, Canada; e British Columbia Emergency Health Services, Vancouver, British Columbia, Canada; f Department of Emergency Medicine, The Ohio State University Wexner Medical Center, Columbus, OH, United States; g Department of Emergency Medicine, Max Rady College of Medicine, University of Manitoba, Winnipeg, Manitoba, Canada; h Division of Emergency Medicine, Department of Medicine, University of Toronto, Toronto, Ontario, Canada; i Emergency Services, Sunnybrook Health Sciences Centre, Toronto, Ontario, Canada; j Department of Critical Care Medicine, Sunnybrook Health Sciences Centre, Toronto, Ontario, Canada; k Interdepartmental Division of Critical Care, University of Toronto, Toronto, Ontario, Canada; l Kingston General Health Research Institute, Kingston Health Sciences Centre, Kingston, ON, Canada; m Department of Critical Care Medicine, Queen’s University, Oshawa, Ontario, Canada; n Department of Critical Care Medicine, Lakeridge Health, Oshawa, Ontario, Canada; o Faculty of Medicine, University of British Columbia, Vancouver, British Columbia, Canada; p Northern Ontario School of Medicine, Sudbury, Ontario, Canada

**Keywords:** Cardiac arrest, Cardiopulmonary resuscitation, Mobile devices, Crowdsourcing, Paramedic services, Randomized controlled trial, Clinical trials

## Abstract

Out-of-hospital cardiac arrest (OHCA) is a leading cause of mortality. Despite public awareness campaigns, widespread cardiopulmonary resuscitation (CPR) training initiatives, and deployment of public access defibrillators, potentially life-saving bystander intervention occurs inconsistently. Mobile technologies that alert nearby lay responders or off-duty professionals to OHCA events have emerged as a strategy to increase bystander CPR and AED use. The PulsePoint Randomized Controlled Trial (RCT) is a multi-centre pragmatic RCT designed to evaluate whether alerts sent via the PulsePoint Respond mobile application increase the likelihood of bystander resuscitation attempts before paramedic arrival.

The trial is being conducted within a province-wide Canadian paramedic service and a municipal fire rescue service in the USA. Eligible 9-1-1 calls for suspected non-traumatic OHCA occurring in a public (non-residential) location are randomized in real time to activation or suppression of the PulsePoint system. The primary outcome is bystander CPR or AED use prior to paramedic or firefighter arrival. Patients are included in the primary analysis if they are determined to have paramedic-treated OHCA in a public location with at least one PulsePoint user within 400 m. The target sample size is 340 patients powered at 80 % to detect a 15 % absolute increase in the primary outcome.

This pragmatic trial addresses a critical evidence gap in resuscitation science. We anticipate findings will inform refinement of technology implementation, policy, guideline development, and system-level decisions regarding the implementation of mobile alert systems to improve early intervention and survival from OHCA.

## Highlights

• First North American RCT to evaluate crowdsourced response for out-of-hospital cardiac arrest (OHCA).
• PulsePoint Respond mobile app alerts aim to boost CPR and AED use before paramedics arrive.
• Employs automated real-time randomization process integrated within 9-1-1 dispatch systems.
• Registry-linked outcomes assess PulsePoint Respond across diverse settings.
• Trial results to guide policy on app-based community responder approaches for OHCA.

## Introduction

Out-of-hospital cardiac arrest (OHCA) is a time-critical emergency affecting approximately 60,000 Canadians and 356,000 Americans annually. Fewer than 10 % survive to hospital discharge.^1^, ^2^ Early cardiopulmonary resuscitation (CPR) and automated external defibrillator (AED) use can increase the odds of survival by two to three times,^3^, ^4^, ^5^, ^6^ but only if these interventions occur rapidly. The median paramedic response time is approximately seven minutes in optimized urban systems and can exceed 14 min in rural areas or hard-to-access urban locations.^7^, ^8^ To mitigate delays in professional response, efforts have been made to encourage bystander CPR and AED use while professionals are en route. Despite sustained global efforts to promote CPR and public access defibrillation, bystander CPR occurs in fewer than 50 % of OHCA cases, and bystander AED use remains below 3 % in many communities.^2^

Community volunteer responder programs using mobile apps, text messages, or other dispatch technologies have emerged as promising strategies to reduce delay to CPR and defibrillation. However, the International Liaison Committee on Resuscitation (ILCOR) has classified the evidence supporting community first responders engaged by technology as “very low-certainty” and has called for clinical trials to generate more robust evidence.^9^ The only published randomized controlled trial (RCT) to date was done in Scandinavia,^10^ highlighting the need for replication in other geographical contexts.

To address the evidence gap, we have designed and implemented a RCT to evaluate the impact of a commonly used mobile device application called “PulsePoint Respond” designed to crowdsource bystander resuscitation for patients who experience OHCA. Our primary objective is to determine whether “CPR Needed” alerts sent via the PulsePoint Respond app during dispatch procedures increase the occurrence of bystander CPR or AED use before professional responders arrive, compared with standard dispatch procedures without PulsePoint alerts. Our secondary objective is to examine how cardiac arrest event characteristics (e.g., initial rhythm, location type, time of day) and system characteristics (e.g., PulsePoint user density) influence the likelihood of bystander resuscitation following a PulsePoint alert.

## Methods

We used the SPIRIT 2025 checklist of items to address in a randomized controlled trial protocol to structure this report (Supplementary Material 1).^11^

### Open science features

The trial website can be found at https://emergencymed.queensu.ca/research/publications-research-studies. This report is based on protocol version 4.0, modified on October 28,2024. The full trial protocol and analysis plan is available from the corresponding author on request. With appropriate research ethics approval, agreement from participating emergency medical services (data owners), and data-sharing agreements de-identified participant data (including data dictionary) and statistical code will be made available on request to the corresponding author.

### Design

This is a pragmatic, parallel-group, superiority randomized controlled trial.

### Setting

This study is being conducted in two locations: British Columbia Emergency Health Services (BCEHS), which serves the Canadian province’s 5.7 million residents across urban, suburban, and rural settings, and the Columbus Division of Fire, which serves a mostly urban population of 900,000 in the U.S. city of Columbus, Ohio.

### Financial support, study registration and ethics review

This project is supported by an operating grant from the Canadian Institutes of Health Research (Partnerships for Health System Improvement program, FRN #148168), and an investigator-initiated grant from ZOLL Medical Corporation. We registered the trial at clinicaltrials.gov on March 16, 2021 (ClinicalTrials.gov ID NCT04806958, https://www.clinicaltrials.gov/study/NCT04806958#study-plan). The protocol was approved by the Queen’s University Health Sciences & Affiliated Teaching Hospitals Research Ethics Board (REB) (TRAQ # 6020246), the University of British Columbia REB (H17-00310), and the Ohio State University Institutional Review Board (IRB)(#2017H0440) with a waiver of the requirement for informed consent.

### Pre-trial community consultation

Formal community consultations were mandated by the Ohio State University IRB to assess community attitudes toward the study design, the waiver of consent, and the overall acceptability of the intervention. In February 2019 we deployed an online survey targeting residents of Columbus and a telephone survey for residents in both British Columbia and Columbus. The online survey garnered 187 responses indicated support for the proposed clinical trial: 97 % of respondents viewed the study as “very important” or “important,” and 95 % reported feeling “very comfortable” to “neutral” with the planned research. Less than 4 % expressed concerns about the waiver of consent. The telephone survey was conducted by a contract survey organization (HR2 Analytics, Bellevue, Washington). In Columbus (*n* = 70), 99 % of respondents reported being “very comfortable”, “comfortable” or “neutral” with the proposed study, and 93 % expressed strong support for its importance. Similarly, in BC (*n* = 30), 97 % were comfortable with the study, and 90 % viewed the research as important. Only 7 % cited privacy concerns, including potential unauthorized sharing of personal health information or real-time location.

### Comparison groups defined and characterization of the control group

The treatment group receives activation of the PulsePoint Respond system in addition to standard emergency medical dispatch procedures. The control group receives standard emergency dispatch procedures only. Both study locations follow established dispatch protocols. BCEHS has three linked provincial communications centers that use Medical Priority Dispatch System (MPDS) protocols to categorize emergencies, trigger telecommunicator CPR instructions, and dispatch ambulances and partnered first‑responder fire units to high‑priority calls such as suspected cardiac arrests. In Columbus, the Division of Fire operates a fire‑medic model with Basic Life Support and Advanced Life Support teams dispatched simultaneously for cardiac arrests, responding from 35 stations equipped with fire apparatus and medic units. Both systems ensure rapid professional response; our trial evaluates whether adding PulsePoint‑activated community responders provides incremental benefit. The study intervention concludes in the prehospital setting. All care provided to participants allocated to either group is at the discretion of the clinicians in the prehospital and hospital settings.

### Characterization of the treatment group: PulsePoint respond

**Fig. 1:**
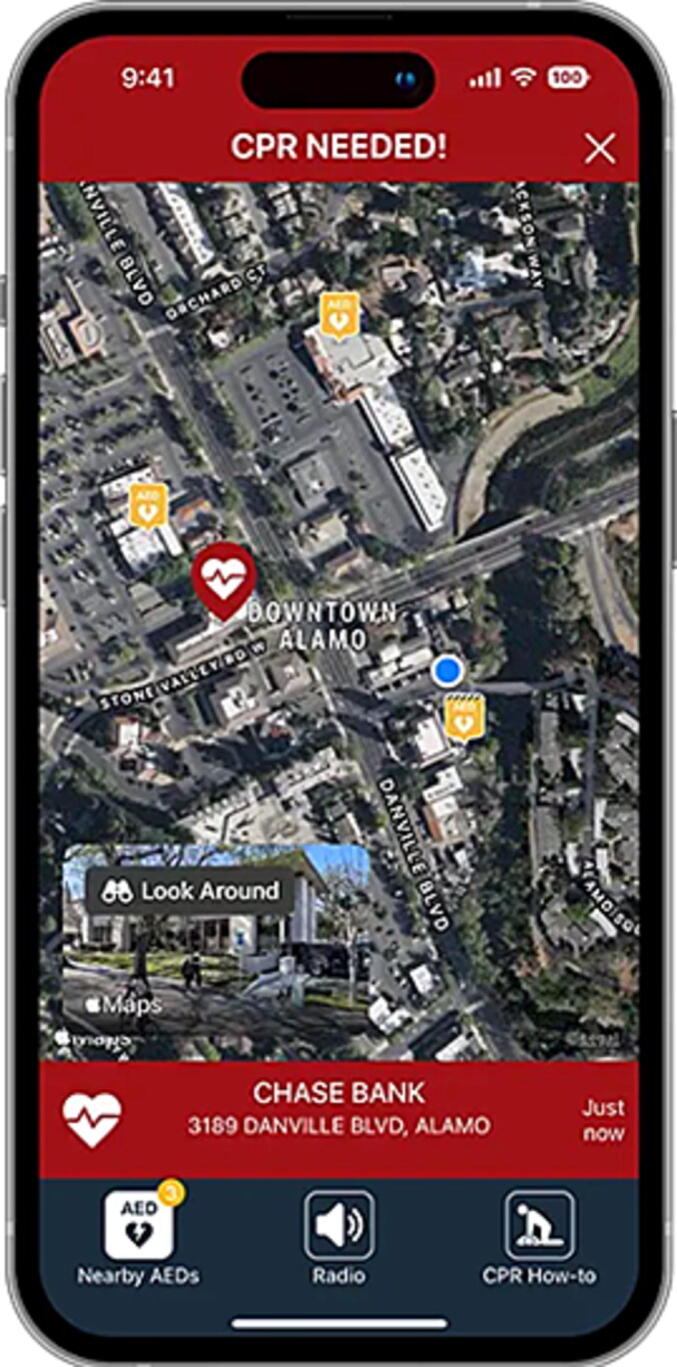
A PulsePoint “CPR Needed” alert with an interactive map showing the location of the user (blue dot) in relation to the location of the emergency (red heart icon) and nearby public access defibrillators (yellow AED icon) (For interpretation of the references to colour in this figure legend, the reader is referred to the web version of this article).

PulsePoint Respond consists of two main components: the PulsePoint interface software and the PulsePoint Respond mobile device application. The interface software is installed on computer-assisted dispatch (CAD) systems used by emergency call-takers to manage emergencies and coordinate paramedic dispatch. The mobile device app is downloaded by community volunteers onto their own mobile phones. During a 9-1-1 call, the PulsePoint interface software screens incident data in real time. If the call is coded on the local CAD as a suspected cardiac arrest in a public location, the PulsePoint system is activated. It then sends “CPR Needed” notifications to nearby users of the PulsePoint Respond app who have opted in to receive these type of alerts and are within approximately 400 m of the incident. Unless users opt out, the notification overrides “Do Not Disturb” settings and plays an audible alarm even when the device is muted. Once acknowledged, it opens an interactive map showing the incident location and nearby public access AEDs (Fig. 1). The PulsePoint notification process operates automatically and does not require the 9-1-1 call-taker to activate the system. To facilitate community uptake, both study sites launched communication strategies, including websites, press releases, media interviews, and social media campaigns aiming to maximize PulsePoint Respond app downloads. We engaged healthcare and resuscitation organizations including CPR training groups, responder unions, public defibrillation charities, and life-saving equipment companies, to promote app downloads amongst members.

### Automated patient recruitment and randomization

Inclusion criteria for the study are 1) 9-1-1 calls assigned as “suspected” or “confirmed” OHCA, and 2) OHCA confirmed as paramedic-treated, public location OHCA. Exclusion criteria include 1) a traumatic cause of cardiac arrest, 2) cardiac arrest occurring in the context of a dangerous scene as determined by the 9-1-1 call-taker, 3) paramedic-witnessed cardiac arrest, 4) cardiac arrest not treated by paramedics (Do Not Resuscitate or signs of obvious death), 5) cardiac arrest occurring in nursing homes. To operationalize these criteria and facilitate the recruitment of OHCA patients at the time of the 9-1-1 call, we undertake the following processes. All 9-1-1 calls coded as possible OHCA incidents occurring in non-residential locations in the computer-assisted dispatch systems of participating communities are automatically enrolled into the trial and undergo random allocation to a treatment group by the PulsePoint server. In BC, Medical Priority Dispatch System codes 09D01 (ineffective breathing), 09E01 (respiratory arrest), 09E02 (uncertain breathing status), 11D01 (choking with altered level of consciousness), 11D02 (choking with abnormal breathing), 11E01 (verified choking), 12D01 (convulsions with absent breathing), and 14D01 (unconscious drowning) define possible OHCA. In Columbus, Association of Public-Safety Communications Officials (APCO) call designators “Cardiac Arrest”, and “Drowning” are used. When the trial was launched, Columbus had also included “Unconscious Overdose”, and “Unconscious Person”, but these codes were later removed from our randomization trigger criteria due to very low specificity for OHCA. Random allocation occurs using code embedded within the PulsePoint server activation process for “CPR Needed” alerts. The code references a randomization table generated by the study statistician enabling 1:1 random allocation, stratified by site, using permuted blocks of varying sizes. For each eligible 9-1-1 call, the next entry in the randomization table determines group assignment. If allocated to the treatment group, the PulsePoint activation proceeds as designed. If assigned to the control group, the process stops before any “CPR Needed” notifications are sent. Each allocation is automatically logged by the server and used by study staff to track enrollment.

### Primary analysis eligibility and case confirmation

**Fig. 2:**
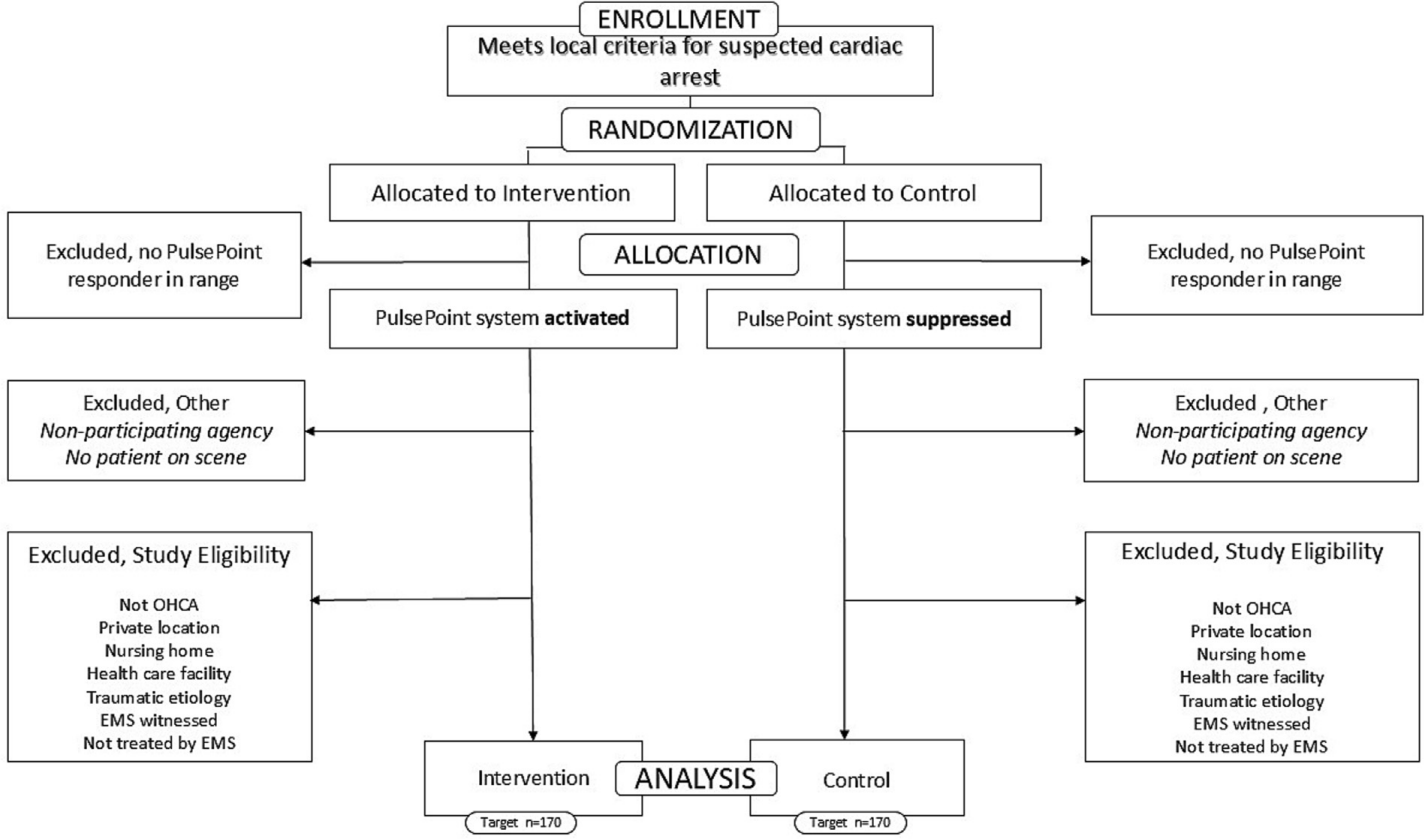
CONSORT diagram showing the enrollment, randomization, and application of inclusion and exclusion criteria to define the primary analysis cohort in the PulsePoint randomized controlled trial.

Randomized patients will be considered eligible for the primary analysis only if they satisfy inclusion criteria, meet no exclusion criteria, and also have at least one PulsePoint responder within 400 m at the time of the 9-1-1 call. Because the intervention and its intended effect must occur before paramedic arrival, randomization must also occur before patient eligibility for the primary analysis can be determined. Study staff retrospectively review each randomized case to determine whether it meets criteria for inclusion in the primary analysis. This approach allows rapid randomization but will introduce many post-randomization exclusions (Fig. 2).

We use pre-existing out-of-hospital cardiac arrest registries to determine eligibility and to capture patient, event, response, and outcome data. Cases from British Columbia are linked to the BC Cardiac Arrest Registry,^12^ and those from Columbus are matched to the CARES registry.^13^ Matching is based on a unique agency incident number, date, time, and location. If no match is found within six months of the incident date, the case is coded as non-OHCA and is excluded. Both registries define cardiac arrest as cases involving CPR or defibrillation by paramedics, or a shock delivered by a bystander AED before their arrival and use validated methods to ensure complete case capture within their regions.

An important determinant of primary analysis eligibility is whether paramedics-initiated resuscitation. Only paramedic-treated cardiac arrests are included, so local protocols guiding initiation of treatment may influence post-randomization exclusions and introduce selection bias. In British Columbia, paramedics initiate CPR unless a valid “No CPR” directive or signs of obvious death (e.g., decapitation, decomposition, rigor mortis) are present. Termination requires online medical control and completion of a structured assessment verified by two providers. In Columbus resuscitation is withheld under a “Dead on Arrival” protocol for patients with irreversible signs of death including decomposition, rigor mortis, dependent lividity, or extreme mottling. Termination may occur after 20 min of advanced life support without ROSC in non-shockable rhythms and requires online medical control consultation and detailed documentation.

### Blinding

The entire randomization and allocation process occurs with blinding of 9-1-1 callers, 9-1-1 call-takers, responding paramedics and firefighters, bystanders, patients, investigators, and the study statistician. Each randomization event is automatically logged by the system and labeled only as “Group A” or “Group B” to preserve allocation concealment. A key, linking group labels to intervention status, is maintained by an independent statistician who will only unblind the study groups after analysis is complete.

### Outcome measures

Outcome selection and reporting are consistent with the Utstein Cardiac Arrest Registry Template.^14^ The primary outcome is bystander resuscitation attempt, defined as the occurrence of either bystander CPR or bystander AED use prior to the arrival of professional emergency responders. Secondary outcomes include bystander CPR, bystander AED use (attachment of pads to the chest), bystander AED shock delivery, return of spontaneous circulation, survival to hospital discharge, and survival to hospital discharge with good functional outcome (Cerebral Performance Score 1 or 2). We planned to collect and report modified Rankin Score, but later discovered that this measure is not available in the cardiac arrest registries. Secondary safety outcomes include paramedic response time interval, on-scene time interval, and the proportion of patients receiving bystander interference with the resuscitation effort (determined from emergency medical service crew safety reports and primary documentation in the clinical record). Secondary system performance outcomes include the number of PulsePoint application downloads for each agency over the course of the trial, the number of PulsePoint application users notified for each call, the sensitivity of PulsePoint activation calculated as the proportion of OHCA during the trial resulting in a study allocation event, and the false positive rate for PulsePoint activation calculated as the proportion of PulsePoint activations associated with non-cardiac arrest incidents.

### Sample size considerations

We calculated our target sample size to detect a 15 % absolute increase in bystander resuscitation attempts between treatment groups. The study steering committee determined this to be the minimal clinically important difference through consensus. Using updated simulations assuming a consistent odds ratio across strata with varying baseline event rates, we initially targeted a total sample size of 522 patients to achieve 90 % power, assuming a two-sided alpha of 0.05. However, partway through the trial, we revised the target sample size due to slower-than-anticipated recruitment, limited funding, and concerns about the feasibility of an extended study duration. To address these constraints, we reduced the target power to 80 %, yielding a revised sample size of 340 patients (170 per group). We did not account for missing patients in our revised calculations because recruitment data at the time indicated <1 % loss to follow-up.

### Data collection

While the trial does not meet all criteria of a classic registry-based randomized controlled trial (e.g., registry-based randomization), it reflects key elements of the model by enabling efficient capture of rich, structured data through linkage with established registries. Both registries (BC Cardiac Arrest Registry and CARES) draw data from electronic ambulance call reports, fire service records, and hospital charts. They include patient demographics, timestamped prehospital interventions, and outcomes such as survival and neurological status at discharge. Both registries have employed robust data quality measures that have been reported elsewhere.^13^, ^15^ Operational data related to PulsePoint notifications are obtained directly from the PulsePoint server. For each randomized incident, data captured in the PulsePoint server include the date, time, and location of the event, randomization assignment, number and location of app users within the activation radius, and the proximity of the nearest registered public access AED. PulsePoint also provides jurisdiction-level metrics on mobile app uptake and penetration over time, including the number and geographic distribution of active app users. These data will be used to assess both the reach of the intervention and its implementation fidelity. De-identified data from the cardiac arrest registries, depending on case location, along with PulsePoint system data, are securely transferred to the study coordinating centre at Queen’s University. All data are stored in a dedicated REDCap (Vanderbilt University, Nashville, TN) instance hosted within the Centre for Advanced Computing at Queen’s University. The platform resides on secure, password-protected research server that adheres to recognized international standards for research data privacy and security, including physical and electronic safeguards consistent with ethical and legal requirements for health data management in both Canada and the United States. Data transferred and stored for the purposes of this study are de-identified and do not contain direct personal identifiers such as name, date of birth, or health card number. Each case is linked using a unique emergency service incident number, allowing case matching without the transfer of identifiable information into study databases.

### Safety

A comprehensive safety monitoring framework is embedded within the trial protocol. Adverse events and serious adverse events have been defined a priori, with specific criteria established to differentiate between events requiring clinical escalation and those involving scene dynamics or responder behavior. Examples of reportable serious adverse events include injury or death to a bystander, grossly negligent CPR resulting in harm, or violent altercations requiring medical intervention. Less severe but still reportable events include vandalism, property theft, non-injurious physical altercations, and scene overcrowding that impedes paramedic operations. Data sources for safety surveillance include structured abstraction from paramedic patient care records, quality improvement systems, and ongoing engagement with paramedic leadership. In addition, the trial employs routine safety check-ins at steering committee meetings and issues regular reminders to frontline personnel and data abstractors to facilitate early identification and documentation of any safety-related concerns.

### Analysis plan

The primary outcome will be compared between treatment groups using the Mantel-Haenszel Chi-square test, stratified by site. Estimates of the treatment effect will be reported as absolute risk differences and relative risks, each accompanied by 95 % confidence intervals. Binary secondary outcomes will be analyzed using the same stratified approach. Continuous outcomes, such as emergency services response time and on-scene time intervals, will be analyzed using the stratified Wilcoxon rank-sum (van Elteren) test to account for non-normal distributions while stratifying by site.

The secondary objective will be addressed using a hierarchical logistic regression model to examine factors associated with the receipt of bystander resuscitation following PulsePoint notification. This model will account for clustering by site and include independent variables such as age, sex, witnessed status, time of day, day of the week, paramedic response interval, number of PulsePoint users in proximity, and whether dispatcher-assisted CPR instructions were provided. Results will be expressed as adjusted odds ratios with 95 % Wald confidence intervals. The same modeling approach will be applied to selected binary secondary outcomes. Subgroup analyses will examine whether the effect of the intervention differs across key subpopulations. These will include a comparison of events in rural versus urban settings, as defined by Statistics Canada census metropolitan area classifications, and a comparison of cardiac arrests with witnessed shockable rhythms versus non-shockable rhythms. Interaction terms will be included in regression models to formally test for effect modification.

## Knowledge dissemination

Results from this trial will be communicated through peer-reviewed publication, scientific conference presentations, posting on ClinicalTrials.gov. A plain language summary will be developed and shared via institutional websites and social media. We will also engage paramedic services, public health units, and CPR advocacy organizations to share findings with community members, lay responders, and healthcare professionals in participating regions.

## Trial management

The trial is coordinated from Queen’s University in Kingston, Ontario. Oversight is provided by a steering committee comprising of the principal investigator, lead research coordinator, co-investigators, technical representatives from participating sites, and research personnel responsible for local implementation and data collection. The committee meets bi-monthly to monitor study progress, review reports of adverse events, and address operational challenges. An independent Data and Safety Monitoring Board (DSMB) is guided by a charter to provide external oversight. DSMB members have expertise in clinical trials, resuscitation science, paramedic service operations, paramedicine, and emergency medicine. The DSMB meets every six months to review maintenance of equipoise, patient safety, enrollment rates, protocol adherence, and data quality. Within 48 h after each meeting, the DSMB provides a report to the principal investigator recommending continuation, modification, or termination of the study. A copy of the DSMB charter can be obtained from the corresponding author. Protocol modifications are reported to all site PIs, the DSMB, and all relevant research ethics boards.

## Trial progress to date

The trial launched on June 8, 2021 in Columbus and July 30, 2021 in British Columbia. As of May 12, 2025, a total of 288 patients have been successfully enrolled and included in the primary analysis dataset. We anticipate completing enrollment by December 1, 2025.

## Discussion

Crowdsourcing applications like PulsePoint Respond, which alert nearby lay responders to suspected cardiac arrests, offer a novel approach to improving early resuscitation. While observational studies and system-level evaluations suggest possible benefits^16^, ^17^, ^18^, ^19^, ^20^, ^21^, ^22^, ^23^, ^24^, ^25^, ^26^, ^27^, ^28^, ^29^, ^30^, evidence from randomized trials remains limited. Only one published RCT has evaluated crowdsourcing technology for OHCA, but it involved a text-message based intervention in a Scandinavian setting, included private residential locations, was only active during the daytime, and did not involve notification of nearby public AED locations.^10^ The benefit observed in this trial was encouraging, but modest. The intervention increased bystander CPR by 13.9 percentage points without any significant increase in survival.

Despite the paucity of data from clinical trials, this approach has proliferated. We conducted a scoping review of the literature which identified an 25 different crowdsourcing technologies being used in 15 countries around the globe.^31^ At the time of writing, PulsePoint has been implemented in over 5300 U.S. communities and has more than 1.3 million app users.^32^ This widespread adoption without more evidence from clinical trials is problematic. Observational studies such as pre-post comparisons or analyses of alerted vs. non-alerted cases, are prone to significant bias. Bystander CPR rates could increase over time due to unrelated public health initiatives, and alerts tend to occur in densely populated areas where circumstantial bystanders are more likely regardless of the app. These factors could result in an overestimate of the true impact of the technology.

In this context, clinical equipoise remains due to uncertain benefit, associated costs, and potential risks of crowdsourcing technology. Agencies adopting the technology incur expenses related to software licensing and the human resources required for program management, and ongoing public engagement. Theoretical risks include potential injuries to responders en route, liability issues for hosting agencies, unsafe scene conditions for responders, and risks to patient safety through poor-quality CPR, privacy breaches, and theft. These uncertainties underscore the need for high-quality evidence from clinical trials to determine whether the benefits of crowdsourcing technologies for OHCA justify their costs and potential risks.

This has been a very challenging trial to implement and manage. Of the five originally proposed study sites, only two were ultimately able to participate. Concerns related to legal liability for the hosting agency, specifically around the randomization of cardiac arrest patients and the sharing of patient data prevented participation of proposed sites in Toronto, Oshawa, and Winnipeg. Despite several years of engagement with these sites and successful technical implementation of the PulsePoint system we were unable to reach a resolution on these issues and efforts to implement the trial in these places were abandoned.

Technical hurdles contributed to significant delays. In British Columbia, accurately distinguishing public from private locations during 9-1-1 calls was essential for determining trial eligibility, as the PulsePoint Respond app was initially designed to notify for public-location arrests only. To support public-only alerts, the Foundation developed custom geolocation coding for BC, and delays in its development, implementation, and testing postponed the site’s launch by several months. The COVID-19 pandemic introduced disruption and delays to trial implementation. Concerns about infection risk among lay responders, paramedics, and firefighters led to a delayed launch of the study for nearly a year. After launch, both active study sites experienced intermittent downtime due to cybersecurity threats, CAD software upgrades, and technical malfunctions. Even after all these issues were resolved, recruitment has been slower than projected, primarily due to limited public uptake of the PulsePoint app and the restrictive inclusion criteria necessary for a scientifically rigorous evaluation. A key contributor to this challenge was the lack of dedicated funding for community outreach, marketing, or communications. The study budget did not include resources for promotional activities, and the hosting agencies, while very supportive, had limited capacity to fund or lead sustained public engagement campaigns. As a result, all efforts to promote PulsePoint uptake in participating communities were conducted in-kind, using low-cost strategies.

Our methodology has some limitations. We are using a modified intention-to-treat approach which includes only paramedic-treated cardiac arrests in the analytic cohort. Because paramedic resuscitation must be initiated to meet inclusion criteria, the presence of a bystander responder, especially one delivering CPR, could influence paramedic decision-making and increase the likelihood of paramedic treatment. This could lead to differential post-randomization exclusion between groups and introduce selection bias. Importantly, the direction and magnitude of this bias depend on the true effect of the PulsePoint intervention. If PulsePoint increases bystander CPR, more cases in the intervention group may meet inclusion criteria, potentially exaggerating the observed effect. If PulsePoint has no impact, exclusions may be more balanced, and bias minimized. Both participating paramedic services follow structured, protocol-driven criteria to guide treatment decisions, including defined indicators for withholding or terminating resuscitation, which helps to reduce subjective variation. Nonetheless, the potential for selection bias due to pre-eligibility randomization remains and will be considered when interpreting trial findings.

This trial addresses the urgent need for innovation in OHCA response. Bystander CPR and AED use remain far too low, and the status quo is not acceptable. We pursued this complex public intervention because new strategies must be evaluated with scientific rigor. Despite significant challenges, this study is uniquely designed, with automated real time randomization and blinding integrated into dispatch systems. Its findings will provide rare and valuable evidence to guide future policy, innovation, and refinement of this promising new approach to OHCA.

## Funding sources

This trial was funded by a Partnerships for Health Systems Improvement grant from the 10.13039/501100000024Canadian Institutes of Health Research (FRN 148168), and an Investigator-initiated research grant from ZOLL Medical Corporation. The sponsors were not involved in, and had no authority over the study design, collection, analysis and interpretation of data, writing of the report or decision to submit the article for publication.

## Declaration of Generative AI and AI-assisted technologies in the writing process

During the preparation of this work the authors used ChatGPT to improve the readability and language of the manuscript. After using this tool, the authors reviewed and edited the content as needed and take full responsibility for the content of the published article.

## CRediT authorship contribution statement

**Steven C. Brooks:** Writing – review & editing, Writing – original draft, Supervision, Project administration, Methodology, Funding acquisition, Conceptualization. **John M. Tallon:** Writing – review & editing, Conceptualization, Methodology, Funding acquisition. **Sandra Jenneson:** Writing – review & editing, Conceptualization, Methodology, Resources, Project administration, Funding acquisition. **Ashish R. Panchal:** Writing – review & editing, Conceptualization, Methodology, Resources, Project administration, Funding acquisition. **Robert Grierson:** Writing – review & editing, Resources, Project administration, Funding acquisition. **Laurie J. Morrison:** Writing – review & editing, Conceptualization, Methodology, Funding acquisition. **Damon C. Scales:** Writing – review & editing, Conceptualization, Methodology, Funding acquisition. **Andrew Day:** Writing – review & editing, Methodology, Formal analysis, Funding acquisition. **Lindsay O’Donnell:** Writing – review & editing, Project administration, Data curation. **Randy S. Wax:** Writing – review & editing, Conceptualization, Methodology, Project administration, Funding acquisition. **Helen Connoly:** Writing – review & editing, Project administration, Investigation. **Jennie Helmer:** Writing – review & editing, Methodology, Project administration, Resources. **Heidi Corneil:** Writing – original draft, Writing – review & editing. **Jim Christenson:** Writing – review & editing, Conceptualization, Methodology, Project administration, Investigation, Resources, Supervision, Project administration, Funding acquisition.

## Declaration of competing interest

The authors declare the following financial interests/personal relationships which may be considered as potential competing interests: SCB is the Chief Medical Officer for Rapid Response Revival an AED manufacturer based in Australia. He owns equity in this company. He receives research grant funding from ZOLL Medical Corporation. He receives in-kind research support from SaveStation, and GoodSAM. JMT, SJ, ARP, RG, LJM, DCS, AD, LO, RSW, HC, JH, HC, and JC do not have any declarations of competing interest.
