# Supplementary material for: Protocol for a parallel-group, superiority randomized controlled trial of the PulsePoint mobile application to increase bystander resuscitation in out-of-hospital cardiac arrest
Source: Resusc Plus. 2025 Jul 24;25:101036. doi: 10.1016/j.resplu.2025.101036 (PMC12365335; doi:10.1016/j.resplu.2025.101036)
Supplement: Supplementary Data 2 [file mmc2.docx]

Structured Trial Summary With Items from the WHO Trial Registration Data Set

**Primary Registry and Trial Identifying Number**

ClinicalTrials.gov ID NCT04806958

**Secondary Identifying Numbers**

None

**Source(s) of Monetary or Material Support**

Partnerships for Health Systems Improvement grant from the Canadian Institutes of Health Research (FRN 148168)

Investigator-initiated research grant from ZOLL Medical Corporation.

**Primary Sponsor**

Investigator-initiated

**Contact for Public Queries**

Dr. Steven C. Brooks

+1-613-549-6666 X7497

Steven.Brooks@kingstonhsc.ca

**Contact for Scientific Queries**

Dr. Steven C. Brooks

+1-613-549-6666 X7497

Steven.Brooks@kingstonhsc.ca

**Public Title**

The PulsePoint Study

**Scientific title**

Evaluating the PulsePoint Mobile Device Application to Increase Bystander Resuscitation for Victims of Sudden Cardiac Arrest

**Countries of Recruitment**

Canada

United States of America

**Health Condition(s) or Problem(s) Studied**

Out-of-hospital cardiac arrest

**Intervention(s)**

Activation of the PulsePoint mobile device application system to crowdsource basic life support for patients with out-of-hospital cardiac arrest

**Key Inclusion and Exclusion Criteria**

A priori inclusion criteria for the study are 1) 9-1-1 calls assigned as “suspected” or “confirmed” OHCA, and 2) OHCA confirmed as paramedic-treated, public location OHCA. Exclusion criteria include 1) a traumatic cause of cardiac arrest, 2) cardiac arrest occurring in the context of a dangerous scene as determined by the 9-1-1 call-taker, 3) paramedic-witnessed cardiac arrest, 4) cardiac arrest not treated by paramedics (Do Not Resuscitate or signs of obvious death), 5) cardiac arrest occurring in nursing homes.

**Study Type**

Parallel group, superiority randomized controlled trial

**Date of First Enrollment (planned)**

2021-06-08

**Sample Size**

340

**Primary outcome(s)**

Bystander resuscitation defined as the occurrence of either bystander CPR (chest compressions and or ventilations) or bystander application of a defibrillator prior to the arrival of emergency medical services.

**Key Secondary outcome(s)**

Proportion of patients receiving bystander CPR (secondary effectiveness outcome)

Proportion of patients receiving bystander defibrillator use (secondary effectiveness outcome)

Proportion of patients receiving bystander defibrillator shock delivered (secondary effectiveness outcome)

Proportion of patients with return of spontaneous circulation (secondary effectiveness outcome)

Proportion of patients surviving to hospital discharge (secondary effectiveness outcome)

Proportion of patients surviving to hospital discharge with good functional outcome (Cerebral Performance Category 1 or 2, secondary effectiveness outcome)

EMS response time interval (secondary safety outcome)

EMS on scene time interval (secondary safety outcome)

Proportion of patients receiving bystander interference with the resuscitation effort (secondary safety outcome)

**Ethics Review**

The protocol was approved by the Queen’s University Health Sciences & Affiliated Teaching Hospitals Research Ethics Board (REB) (TRAQ # 6020246), the University of British Columbia REB (H17-00310), and the Ohio State University Institutional Review Board (IRB)(#2017H0440) with a waiver of the requirement for informed consent.

**Individual Trial Participant Data sharing statement**

No. Individual participant data will not be shared.
